# Antiretroviral Therapy Initiation Before, During, or After Pregnancy in HIV-1-Infected Women: Maternal Virologic, Immunologic, and Clinical Response

**DOI:** 10.1371/journal.pone.0006961

**Published:** 2009-09-09

**Authors:** Vlada V. Melekhin, Bryan E. Shepherd, Samuel E. Stinnette, Peter F. Rebeiro, Gema Barkanic, Stephen P. Raffanti, Timothy R. Sterling

**Affiliations:** 1 Division of Infectious Diseases, Department of Medicine, Vanderbilt University School of Medicine, Nashville, Tennessee, United States of America; 2 Department of Biostatistics, Vanderbilt University Medical Center, Nashville, Tennessee, United States of America; 3 Comprehensive Care Center, Nashville, Tennessee, United States of America; 4 Center for Health Services Research, Vanderbilt University School of Medicine, Nashville, Tennessee, United States of America; University of Cape Town, South Africa

## Abstract

**Background:**

Pregnancy has been associated with a decreased risk of HIV disease progression in the highly active antiretroviral therapy (HAART) era. The effect of timing of HAART initiation relative to pregnancy on maternal virologic, immunologic and clinical outcomes has not been assessed.

**Methods:**

We conducted a retrospective cohort study from 1997–2005 among 112 pregnant HIV-infected women who started HAART before (N = 12), during (N = 70) or after pregnancy (N = 30).

**Results:**

Women initiating HAART before pregnancy had lower CD4+ nadir and higher baseline HIV-1 RNA. Women initiating HAART after pregnancy were more likely to receive triple-nucleoside reverse transcriptase inhibitors. Multivariable analyses adjusted for baseline CD4+ lymphocytes, baseline HIV-1 RNA, age, race, CD4+ lymphocyte count nadir, history of ADE, prior use of non-HAART ART, type of HAART regimen, prior pregnancies, and date of HAART start. In these models, women initiating HAART during pregnancy had better 6-month HIV-1 RNA and CD4+ changes than those initiating HAART after pregnancy (−0.35 vs. 0.10 log_10_ copies/mL, *P = *0.03 and 183.8 vs. −70.8 cells/mm^3^, *P* = 0.03, respectively) but similar to those initiating HAART before pregnancy (−0.32 log_10_ copies/mL, *P* = 0.96 and 155.8 cells/mm^3^, *P* = 0.81, respectively). There were 3 (25%) AIDS-defining events or deaths in women initiating HAART before pregnancy, 3 (4%) in those initiating HAART during pregnancy, and 5 (17%) in those initiating after pregnancy (*P = *0.01). There were no statistical differences in rates of HIV disease progression between groups.

**Conclusions:**

HAART initiation during pregnancy was associated with better immunologic and virologic responses than initiation after pregnancy.

## Introduction

Women comprise an increasing proportion of HIV-infected persons, and most of these women are of child-bearing age [1]. Although mother-to-child transmission of HIV has been reduced to <2% of HIV-infected pregnancies due to universal prenatal HIV counseling and testing, antiretroviral therapy, scheduled Cesarean delivery, and avoidance of breastfeeding [2]–[4], challenges remain in improving primary prevention and antiretroviral treatment of HIV-infected women. Moreover, the effects of these interventions on maternal HIV disease progression have not been fully assessed.

There are conflicting data in the literature on the effect of pregnancy on HIV disease progression and survival among HIV-infected women. Studies conducted early in the HIV epidemic reported a possible association between pregnancy and accelerated HIV disease progression [5]–[7], particularly in developing countries [8], [9]. However, studies conducted in the United States and Europe did not find a detrimental effect of pregnancy [10]–[14]. These studies were conducted prior to the era of highly active antiretroviral therapy (HAART) and had significant methodological differences that made it difficult to asses the true effect of pregnancy on HIV disease progression [15].

A study conducted by Tai et al of women from our HIV clinic receiving care in the HAART era, found that pregnancy was associated with a lower risk of HIV disease progression [16]. Although the pregnant women were younger and healthier at HAART initiation than the women who did not become pregnant, pregnant women had lower rates of AIDS-defining illnesses and deaths after controlling for age, baseline CD4+ lymphocytes and HIV-1RNA, and durable virologic suppression. The findings persisted after including a propensity score for pregnancy, and in an analysis that matched pregnant and nonpregnant women according to date of cohort entry, baseline CD4+ lymphocyte count, receipt of HAART, and age at study entry. These results suggested a possible beneficial interaction between pregnancy and HAART in HIV-infected women.

However, to our knowledge, no study has examined the response to HAART among HIV-infected women according to timing of HAART initiation in relation to their pregnancy. We hypothesized that women starting HAART during pregnancy would have improved virologic, immunologic, and clinical responses. If proven clinically significant, improved maternal HIV outcomes would indicate the need to conduct similar studies in resource-limited settings. We therefore conducted a retrospective cohort study to evaluate changes in HIV-1 RNA and CD4+ lymphocytes after starting HAART before, during, or after pregnancy, and subsequent HIV disease progression while in care.

## Materials and Methods

### Study cohort

The study cohort was defined as HIV-1-infected women with at least one pregnancy while receiving care (>1 visit) between 1 January 1997 and 31 December 2005 at the Comprehensive Care Center (CCC) in Nashville, Tennessee. Only women who started their first HAART regimen and had HIV-1 RNA and CD4+ lymphocyte measurements <180 days prior to HAART initiation were included in this study. This cohort was drawn from part of a previously described cohort [16].

Clinical data were entered into an electronic medical record by medical providers at the time of the patient encounter, by automated data upload (e.g., laboratory results), or by clinic personnel (e.g., deaths). Laboratory and antiretroviral therapy data (including regimen and start and stop dates) were validated by systematic chart review. The informed consent process was waived in accordance with the Code of Federal Regulations (CFR) 45 CFR 46.116 (d). The study and the waiver of consent were approved by the Vanderbilt Institutional Review Board.

### Definition of study exposure

Study patients were classified according to timing of first HAART initiation. Women were classified as initiating HAART before, during, or after first pregnancy while in care if they started first HAART ≥30 days before estimated date of conception (DOC), <30 days before DOC and ≤30 days after pregnancy event (defined as delivery, miscarriage, or pregnancy termination), or >30 days after pregnancy event, respectively. DOC was determined by last menstrual period and/or fetal ultrasound. The 30-day window before date of conception was used due to uncertainty of the estimated date of conception as determined by last menstrual period [17]–[20]. The 30-day window after date of pregnancy event was used because of continued changes in immunologic parameters seen after pregnancy event that might influence immunologic and virologic responses to HAART [21], [22]. Women who started HAART during pregnancy were presumed to start HAART for prevention of mother-to-child HIV transmission (PMTCT) and not for maternal health if they had no previous opportunistic infections, had nadir CD4+ lymphocyte count >350 cells/mm^3^, and peak HIV RNA level any time prior to HAART initiation while in care <100,000 copies/mL [2].

HAART was defined as regimens of ≥7 days duration that contained two nucleoside reverse-transcriptase inhibitors (NRTI) plus a protease inhibitor (PI), a non-nucleoside reverse-transcriptase inhibitor (NNRTI), or a third NRTI; one NRTI, one PI, plus one NNRTI; 2 PIs plus one NNRTI or one NRTI; or any regimen containing enfuvirtide. Non-HAART antiretroviral therapy (ART) included mono- or dual-NRTI therapy.

### Definition of study outcomes

Outcomes of interest were rates of HIV-1 RNA and CD4+ lymphocyte change during the first 180 days of HAART initiation and HIV disease progression, which was defined as time to AIDS-defining event (ADE) or death at any time after HAART initiation. ADEs were based on the 1993 US Centers for Disease Control and Prevention classification criteria [23], excluding CD4+ lymphocytes <200 cells/mm^3^.

### Study variables

Maternal characteristics such as age, race, baseline CD4+ lymphocyte count and percentage, CD4+ lymphocyte count nadir (the lowest CD4+ lymphocyte count in care prior to pregnancy event), baseline HIV-1 RNA, date of HAART start, hepatitis C virus (HCV) serological status, hepatitis B virus (HBV) infection, history of injection drug use (IDU) as an HIV acquisition risk factor, history of ADE, prior use of non-HAART ART, date of HAART start, and date of conception, and data on prior (i.e., prior to the study period) and subsequent (i.e., after the first pregnancy while in care during the study period) pregnancies were assessed.

### Study period

Baseline (time 0) was defined as the date of initiation of first HAART. For the analysis of HIV-1 RNA and CD4+ lymphocyte responses to first HAART, the study period ended when one of the following conditions were met: 1) 180 days after first HAART start (to measure early changes in HIV-1 RNA and CD4+ lymphocyte responses); 2) change in pregnancy status; 3) death; 4) 31 December 2005; 5) discontinuation of first HAART regimen; 6) last clinic encounter date if before 31 December 2005. Change in pregnancy status was defined as 30 days before DOC for women initiating HAART before pregnancy and 30 days after pregnancy event for women initiating HAART during pregnancy.

For the time to ADE or death analysis the study period ended with the first ADE or death, last clinic encounter, or 31 December 2005. All events were reviewed and confirmed by study investigators (PFR, GB, SPR).

### Statistical analyses

STATA SE (version 9.2; Stata Corporation) was used for all analyses. Continuous variables were compared with the Kruskal-Wallis test. Categorical variables were compared with the Fisher's exact test.

### HIV-1 RNA and CD4+ lymphocyte analyses

Linear mixed effects models [24] were used to calculate the rates of HIV-1 RNA and CD4+ lymphocyte change according to timing of first HAART initiation (before, during, or after pregnancy). We fit models which adjusted for baseline CD4+ lymphocytes, baseline HIV-1 RNA, age, race, CD4+ lymphocyte count nadir, history of ADE, prior use of non-HAART ART, type of HAART regimen, prior pregnancies, and date of HAART start. These variables were chosen for inclusion in the analyses because of their clinical significance. From these models, we derived the rates of HIV-1 RNA and CD4+ lymphocyte change from interaction terms between time and HAART initiation category (before, during, or after pregnancy). We fit models that did not assume a linear relationship between CD4+ lymphocytes, HIV-1 RNA and time using restricted cubic splines. There was insufficient evidence to suggest that these nonlinear models improved the fit of the data (*P>*0.05 for all tests), so we report only the results of the linear models.

We also performed secondary analyses of HIV-1 RNA and CD4+ lymphocyte change including variables for HCV sero-status and history of IDU on the subset of individuals for whom this information was available. We examined the sensitivity of our analyses by assuming that persons with missing HCV serological status data and IDU data all had or did not have these conditions. Due to the concern that differences in HIV-1 RNA detection limits (400 and 50 copies/mL) during the study period could explain the differences in HIV-1 RNA change between the groups, we also performed sensitivity analyses assuming that every HIV-1 RNA value of <400 copies/mL was equal to 399 copies/mL instead of actual number of virions for HIV-1 RNA level between 50 and 399. Additional analyses of HIV-1 RNA and CD4+ lymphocyte changes repeated the above analyses excluding women on triple-NRTI HAART regimens, since such regimens are now not recommended despite their prior widespread use [25]–[27]. Additionally, we repeated the above analyses after grouping subjects based on DOC and date of pregnancy event without the 30-day window periods.

### HIV disease progression analyses

Cox proportional hazards models compared rates of HIV disease progression (ADE/death) according to timing of HAART initiation, both unadjusted and adjusted for the propensity of belonging to a particular HAART initiation category (before, during, or after pregnancy). Propensity scores [28] for starting first HAART before or after pregnancy versus during pregnancy were derived from separate logistic regression models including age, race, baseline CD4+ lymphocyte count and HIV-1 RNA level, CD4+ lymphocyte count nadir, use of non-HAART ART, type of HAART regimen, prior pregnancies, and date of conception. We were unable to use history of IDU and history of ADE because these variables perfectly predicted HAART initiation groups.

We performed secondary analyses comparing rates of HIV disease progression using HCV sero-status as an additional covariate on the subset of individuals who had this information available, and sensitivity analyses assuming that persons with missing HCV sero-status data all had or did not have HCV infection. We also repeated analyses excluding women on triple-NRTI HAART regimens. Additionally, we repeated the above analyses by grouping subjects based on DOC and date of pregnancy event without the 30-day window periods.

## Results

### Patient characteristics

**Figure 1:**
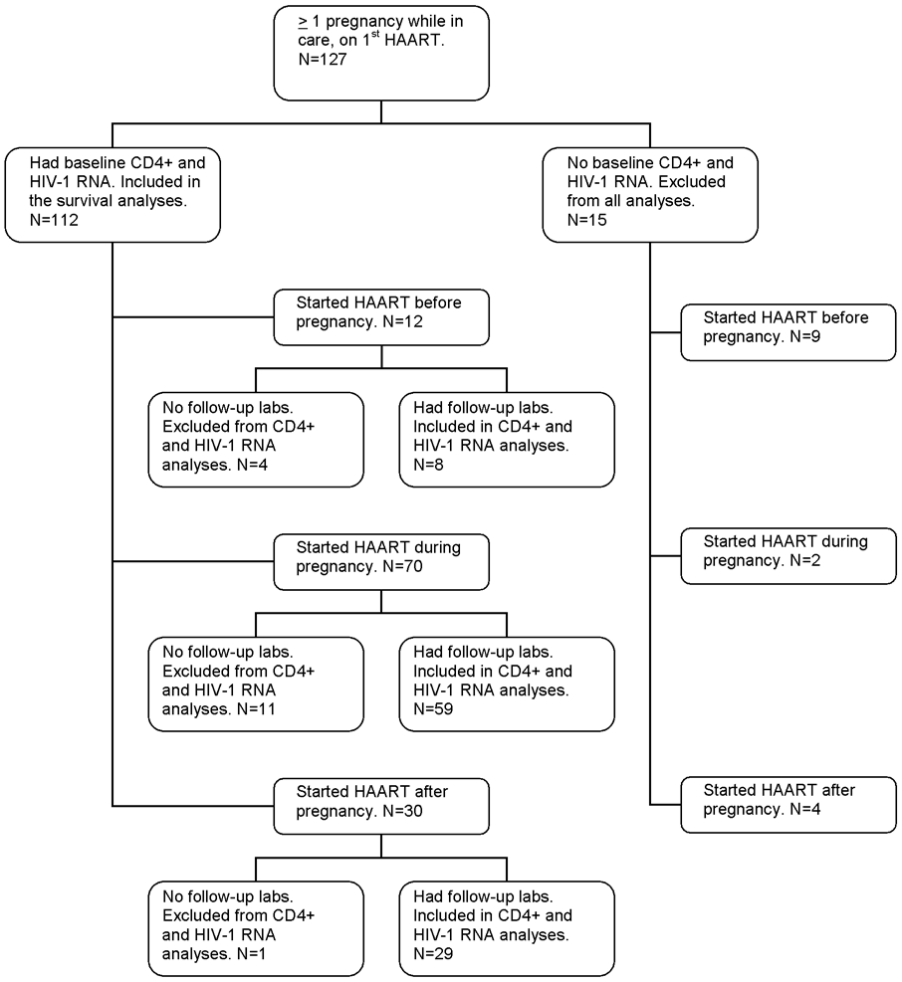
Flow chart for patient selection. HAART: Highly active antiretroviral therapy.

Of the 127 women who were pregnant and initiated their first HAART while followed at the Comprehensive Care Center during the study period, 112 had baseline HIV-1 RNA and CD4+ lymphocytes and were included in the HIV disease progression analysis: 12 women started HAART before pregnancy, 70 women started HAART during pregnancy, and 30 women started HAART after pregnancy. Of the 112, 96 additionally had follow-up labs available during the first 180 days after HAART start and were included in the analysis of HIV-1 RNA and CD4+ lymphocytes following HAART initiation (Figure 1). Fifteen women who did not meet inclusion criteria were similar to the study subjects according to age, HAART duration, and proportion with ADE or death. The 16 (of 112) women without follow-up labs were similar to the study subjects according to age, baseline CD4+ lymphocytes, baseline HIV-1 RNA, HAART duration, and proportion with ADE or death while in care.

Table 1 shows demographic and clinical characteristics of the 112 patients. The groups were similar according to age and race. Women starting HAART before pregnancy had lower CD4+ lymphocyte count nadir, higher baseline HIV-1 RNA, higher proportion with a history of ADE, started their first HAART at an earlier date, were more likely to receive PI-based HAART, and had a longer study period for ADE and death analyses, than women who started HAART during or after pregnancy. Of those with available data, rates of HCV infection and history of IDU were similar across groups. A higher proportion of women who started HAART after pregnancy received HAART regimens other than PI- or NNRTI-based HAART (all were triple-NRTI-based HAART regimens). They also were more likely to have received non-HAART ART prior to HAART and conceived at an earlier date compared to the other two groups. There were no cases of HBV infection.

**Table 1:** Demographic and clinical characteristics of the study population (N = 112).

Characteristic	Started HAART before pregnancy N = 12	Started HAART during pregnancy* N = 70	Started HAART after pregnancy N = 30	*P* a
Age at first HAART start, median (IQR), years	26.5 (23.5–35)	25.5 (22–29)	26 (23–29.5)	0.42
Black race, no. (%)	5 (41.7)	36 (51.4)	12 (40.0)	0.56
Baseline CD4+ lymphocyte count, median (IQR), cells/mm^3^	284 (50–472)	407 (306–494)	430 (144–515)	0.27
Baseline CD4+ lymphocyte percentage, median (IQR), percent	23 (10–34.5)	27 (21–33)	26 (14–34)	0.50
CD4+ lymphocyte count nadir, median (IQR), cells/mm^3^	220 (37–414)	381 (272–465)	284 (130–416)	0.009
Baseline HIV-1 RNA level, median (IQR), log_10_ copies/mL	4.97 (4.20–5.17)	3.89 (3.37–4.49)	3.70 (2.97–4.58)	0.006
Prior ADE, no. (%)	3 (25.0)	1 (1.4)	1 (3.3)	0.01
HCV, no. (%)	2 (16.7)	4 (6.0)	5 (16.7)	0.14
Missing, no. (%)	0	3 (4.3)	0	
IDU, no. (%)	1 (10.0)	3 (5.6)	5 (16.7)	0.24
Missing, no. (%)	2 (16.7)	16 (22.9)	0	
HAART start date, median (IQR), month/year	07/1999 (08/1998–04/2000)	12/2001 (06/2000–01/2004)	04/2001 (08/1999–02/2003)	0.001
First HAART regimen				0.007
PI-based, no. (%)	7 (58.3)	23 (32.9)	7 (23.3)	
NNRTI-based, no. (%)	3 (25.0)	31 (44.3)	6 (20.0)	
Other, no. (%)	2 (16.7)	16 (22.9)	17 (56.6)	
First HAART duration, median (IQR), months	24.1 (1.8–40.3)	6.7 (4.1–32.7)	11.2 (5.5–26.9)	0.76
Prior non-HAART ART use, no. (%)	4 (33.3)	20 (28.6)	26 (86.7)	<0.001
Used prior to pregnancy, no. (%)	4 (33.3)	20 (28.6)	10 (33.3)	
Used during and/or after pregnancy, no. (%)	–	–	16 (53.3)	
Date of conception, median (IQR), month/year	05/2001 (07/1999–12/2002)	07/2001 (01/2000–08/2003)	08/1998 (10/1997–09/1999)	<0.001
Pregnancy duration, median (IQR), weeks	37.5 (24–38)	38 (37–39)	38 (36–39)	0.05
Number of pregnancies prior to the study period, median (IQR)	1 (0–2.5)	1 (1–2)	2 (1–3)	0.26
Number of subsequent pregnancies during study period, median (IQR)	0 (0–0.5)	0	0 (0–1)	0.13
Number of provider visits during study period/month, median (IQR)	0.50 (0.37–0.54)	0.58 (0.35–0.95)	0.39 (0.34–0.62)	0.08
Study period duration for ADE and death analyses, median (IQR), months	77.5 (60.1–83.1)	39.8 (17.8–57.4)	42.1 (15.0–67.0)	0.001

Note: HAART: highly active antiretroviral therapy. IQR: interquartile range. CD4+ lymphocyte count nadir: the lowest CD4+ lymphocyte count while in care. ADE: AIDS-defining event. IDU: history of injection drug use as a risk factor for HIV infection acquisition. HCV: hepatitis C virologic status prior to first HAART initiation. NA: not available. ART: antiretroviral therapy. PI: protease inhibitor. NNRTI: non-nucleoside reverse transcriptase inhibitor.

* The reference group.

a Continuous data were compared by Kruskal-Wallis test. Categorical data were compared by 2-sided Fisher's exact test.

All women who initiated HAART before pregnancy presented to the Comprehensive Care Center prior to the date of conception, compared to 33% of women who initiated HAART during or after pregnancy (*P*<0.001). Thirty (43%) women who initiated HAART during pregnancy started HAART for PMTCT. Of the 30 women, 19 (63%) stopped HAART within 90 days after pregnancy event. Median (IQR) time between HAART initiation and date of conception among 12 women who initiated before pregnancy was 526 (364–1199) days. Median (IQR) time between date of HAART initiation and pregnancy event among 70 women who initiated HAART during pregnancy was 137 (109–169) days. Among 30 women who initiated HAART after pregnancy, median (IQR) time between a pregnancy event and HAART initiation was 682 (364–1563) days. Finally, there were no cases of mother-to-child transmission of HIV-1.

Among the 96 women included in the longitudinal analysis of HIV-1 RNA and CD4+ lymphocytes following HAART start, the study period (median (IQR)) was shorter for those initiating HAART during pregnancy (135 (88–164) days) compared to women initiating HAART before (180 (129–180) days) and after pregnancy (180 (167–180) days) (*P<*0.001). The most common reason for ending the study period was reaching 180 days of follow-up for women who started HAART before and after pregnancy and end of pregnancy for those who started HAART during pregnancy.

Fifty-nine women initiating HAART during pregnancy had more provider visits per month during the study period for HIV-1 RNA and CD4+ lymphocyte analysis (median 1.76 visits) compared to 8 women initiating HAART before (median 0.58) and 29 women initiating HAART after pregnancy (median 0.65) (*P<*0.001). The median (range) number of HIV-1 RNA measurements was 3 (2–4), 4 (2–9), and 3 (1–6) for those initiating HAART before, during, or after pregnancy, respectively (*P<*0.001). The median (range) number of CD4+ lymphocyte measurements was 3 (2–3), 4 (2–4), and 2 (2–2) for the three groups (*P<*0.001).

### HIV-1 RNA following first HAART start

**Figure 2:**
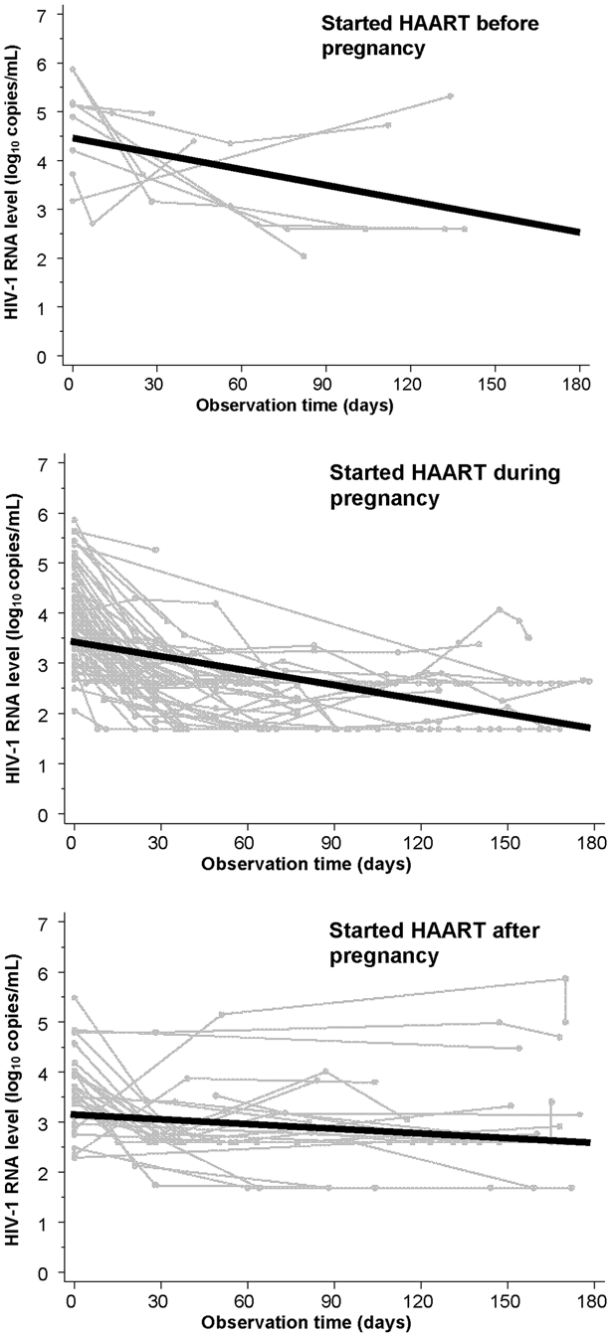
Unadjusted HIV-1 RNA change. Unadjusted HIV-1 RNA following first HAART initiation for each woman (gray lines) and average decline (solid black line) by timing of HAART initiation.

**Figure 3:**
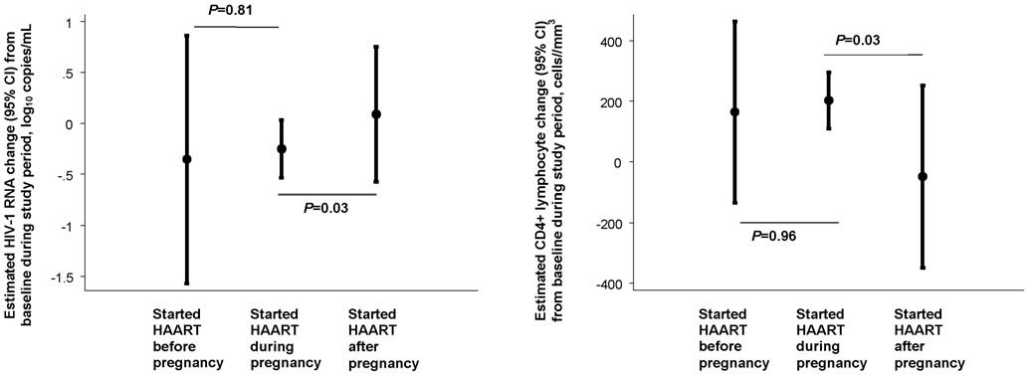
Estimated rate of HIV-1 RNA and CD4+ lymphocyte change. The estimated rate of HIV-1 RNA decline and CD4+ lymphocyte increase (small circles) and 95% confidence interval (vertical bars) by pregnancy group over the 6 months following HAART initiation, adjusted for baseline CD4+ lymphocyte count and HIV-1 RNA, age, race, CD4+ lymphocyte count nadir, prior ADE, prior use of non-HAART ART, HAART type, prior pregnancies, and date of HAART start. Horizontal lines represent *p-*values in a pair-wise comparison (women who started HAART during pregnancy as a reference). Left panel: The estimated rate of HIV-1 RNA decline: −0.32 log_10_ copies/mL (95% CI −1.45, 0.81) in women who started HAART before pregnancy, −0.35 log_10_ copies/mL (95% CI −0.57, −0.13) in women who started HAART during pregnancy, and 0.10 log_10_ copies/mL (95% CI −0.46, 0.66) in women who started HAART after pregnancy. Right panel: The estimated rate of CD4+ lymphocyte increase: estimates were 155.8 cells/mm^3^ (95% CI −107.6, 419.2) in women who started HAART before pregnancy, 183.8 cells/mm^3^ (95% CI 110.8, 256.9) in women who started HAART during pregnancy, and −70.8 cells/mm^3^ (95% CI −326.8, 185.3) in women who started HAART after pregnancy.

Figure 2 shows the unadjusted rate of HIV-1 RNA decline during the study period for all women in each of the three groups. After adjusting for baseline CD4+ lymphocytes, baseline HIV-1 RNA, age, race, CD4+ lymphocyte count nadir, history of ADE, prior use of non-HAART ART, type of HAART regimen, prior pregnancies, and date of HAART start, estimated HIV-1 RNA decline (95% confidence interval (CI)) over the 6 months after HAART initiation for those initiating during pregnancy was similar to that of women initiating prior to pregnancy (*P = *0.96) but greater than that of women initiating after pregnancy (*P* = 0.03): −0.32 log_10_ copies/mL (95% CI−1.45, 0.81), −0.35 log_10_ copies/mL (95% CI −0.57, −0.13), and 0.10 log_10_ copies/mL (95% CI −0.46, 0.66) for women initiating HAART before, during, or after pregnancy, respectively (Figure 3 and Table 2).

**Table 2:** Multivariable linear mixed effects models: independent predictors of HIV-1 RNA levels (log_10_ copies/mL) and CD4+ lymphocyte counts (cells/mm^3^) during 6 months following first HAART initiation* ^&^.

Independent Variables	HIV-1 RNA level predictors, Effect (95% CI)	*P*	CD4+ lymphocyte count predictors, Effect (95% CI)	*P*
Change per month, women who started HAART during pregnancy	−0.06 (−0.09, 0.02)	0.002	30.6 (18.6, 42.9)	<0.001
Interaction term, women who started HAART before pregnancy**	0.03 (−1.03, 1.09)	0.96	−28.1 (−258.5, 202.5)	0.81
Interaction term, women who started HAART after pregnancy**	0.45 (0.5, 0.85)	0.03	−254.5 (−477.0, −32.2)	0.03
Baseline CD4+ lymphocyte count, per 100 cells/mm^3^ increase	0.01 (−0.12, 0.14)	0.86	50.6 (27.0, 74.2)	<0.001
CD4+ lymphocyte count nadir, per 100 cell/mm^3^ increase	−0.10 (−0.24, 0.05)	0.25	72.5 (46.1, 99.0)	<0.001
Baseline HIV-1 RNA level, per log_10_ copies/mL increase	0.24 (0.06, 0.41)	0.01	−0.36 (−31.8, 31.08)	0.98
Age at first HAART start, per year	−0.02 (−0.04, 0.01)	0.25	3.8 (−1.1, 8.8)	0.13
Black race	0.18 (−0.09, 0.44)	0.19	27.9 (−19.7, 75.5)	0.25
Prior ADE (yes/no)	0.58 (−0.27, 1.43)	0.18	−80.9 (−238.7, 76.9)	0.32
Prior non-HAART ART use (yes/no)	0.27 (−0.07, 0.60)	0.16	40.8 (−20.0, 101.5)	0.19
HAART type	0.01 (−0.16, 0.18)	0.87	−5.4 (−36.9, 26.1)	0.74
Prior pregnancies (yes/no)	−0.27 (−0.59, 0.05)	0.10	−46.1 (−104.6, 12.3)	0.12
Date of HAART initiation, per year	−0.0004 (−0.0006, −0.0002)	<0.001	−0.004 (−0.04, 0.03)	0.83

Note: 95% CI: 95% confidence interval. HAART: highly active antiretroviral therapy. CD4+ lymphocyte count nadir: the lowest CD4+ lymphocyte count while in care. ADE: AIDS-defining event. Non-HAART ART: non-HAART antiretroviral therapy.

* Mixed effect model adjusted for baseline CD4+ lymphocyte count and HIV-1 RNA level, age, race, CD4+ lymphocyte count nadir, prior ADE, prior use of non-HAART ART, HAART type, prior pregnancies, and date of HAART initiation.

& The reference group was women who started HAART during pregnancy.

** Interaction terms are equal to the difference in slopes of HIV-1 RNA and CD4+ lymphocyte changes between women who started HAART during pregnancy and those who started HAART before or after pregnancy.

In secondary analyses that included persons in whom HCV serological status and history of IDU data were available, the difference in the estimated HIV-1 RNA decline between those initiating HAART during and after pregnancy was no longer statistically significant: −0.25 log_10_ copies/mL (95% CI −0.53, 0.03) for during and 0.09 log_10_ copies/mL (95% CI −0.57, 0.75) for after (*P* = 0.14). In all other secondary analyses described in the Methods, the estimated HIV-1 RNA decline was greater for those initiating HAART during pregnancy than after (*P*≤0.03 for all analyses, data not shown).

### CD4+ lymphocyte count following first HAART start

**Figure 4:**
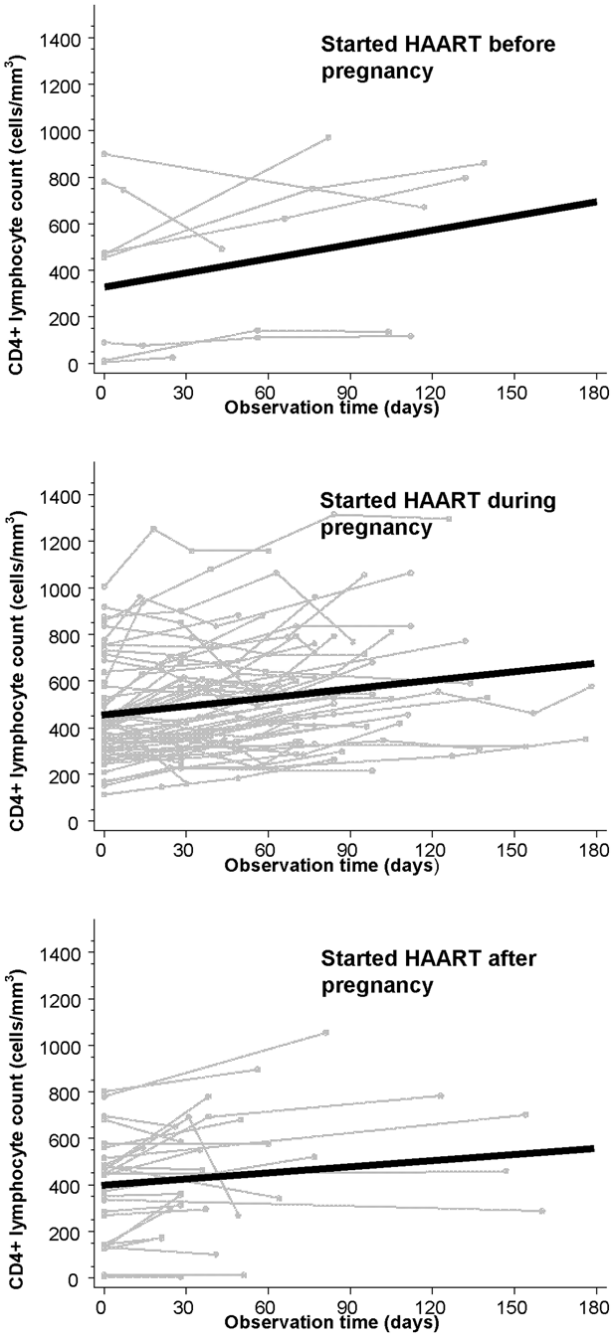
Unadjusted CD4+ lymphocyte count change. Unadjusted CD4+ lymphocyte count following first HAART initiation for each woman (gray lines) and average increase (solid black line) by timing of HAART initiation.

Figure 4 shows the unadjusted rate of CD4+ lymphocyte increase during the study period for all women in each of the three groups. After adjusting for baseline CD4+ lymphocytes, baseline HIV-1 RNA, age, race, CD4+ lymphocyte count nadir, history of ADE, prior use of non-HAART ART, type of HAART regimen, prior pregnancies, and date of HAART start, the estimated rate of CD4+ lymphocyte increase over the 6 months after HAART initiation for women initiating HAART during pregnancy was similar to that of women initiating before pregnancy (*P* = 0.81) and higher than that women initiating after pregnancy (*P = *0.03): 155.8 cells/mm^3^ (95% CI −107.6, 419.2), 183.8 cells/mm^3^ (95% CI 110.8, 256.9), and −70.8 cells/mm^3^ (95% CI −326.8, 185.3) for those initiating HAART before, during, or after pregnancy (Figure 3 and Table 2).

In all secondary analyses (described in Methods), the estimated CD4+ lymphocyte rise was greater for those initiating HAART during pregnancy than those initiating after pregnancy (*P≤*0.04 for all analyses, data not shown).

### HIV disease progression

**Figure 5:**
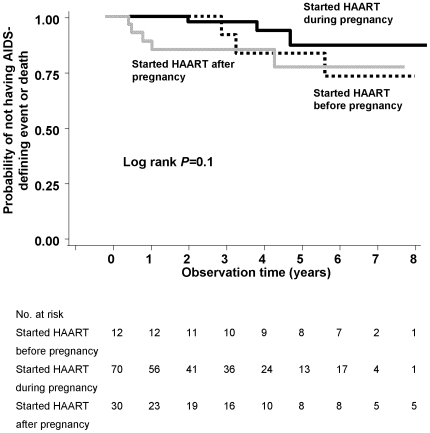
Kaplan-Meier survival curve of progression to new AIDS-defining event or death by timing of HAART initiation. The numbers of women at risk each year are also given.

A lower proportion of women progressed to ADE or death among women starting first HAART during pregnancy compared to those starting HAART before or after pregnancy: 3 (25%), 3 (4%), and 5 (17%) for women who started HAART before, during, and after pregnancy, respectively (*P = *0.01). When women were grouped based on DOC and date of pregnancy event without the 30-day window, the proportion of ADE/deaths did not change between the groups (*P = *0.04). In Kaplan-Meier analysis using the original definition of the groups, the differences in HIV disease progression were not statistically significant (log-rank test, *P = *0.10) (Figure 5). In multivariable Cox proportional hazards models that included propensity scores for initiating HAART before or after pregnancy (as described in the Methods), the hazard of ADE or death did not statistically differ between the groups: compared to those initiating HAART during pregnancy, the hazard ratio (HR) was 1.56 (95% CI 0.17, 14.79; *P = *0.70) for initiating after pregnancy and 0.14 (95% CI 0.01, 3.09; *P* = 0.21) for initiating prior to pregnancy.

Results were similar in secondary analyses that included HCV sero-status in the propensity scores, analyses that excluded women starting triple-NRTI-based regimens, and analyses which grouped subjects based on DOC and date of pregnancy event without the 30-day window (data not shown).

## Discussion

In this study, we compared 6-month changes in HIV-1 RNA and CD4+ lymphocyte and long-term rates of ADE/death in women starting first HAART before, during, or after pregnancy. We found that HIV-1 RNA decline and CD4+ lymphocyte increase were more rapid over the first 6 months among women initiating HAART during pregnancy than after pregnancy. However, there was insufficient evidence to conclude that the rate of disease progression differed according to the timing of HAART initiation relative to pregnancy. In a recent meta-analysis [29], a 0.3 log_10_ increase in HIV-1 RNA was associated with a 25% increase in progression to ADE. Therefore, the difference in virologic response that we noted could potentially have clinical significance. A study with a larger sample size and longer follow-up is needed to assess for significant difference in clinical outcomes.

The etiology of the improved virologic and immunologic responses associated with HAART initiation during pregnancy is likely multifactorial. The improved response could be due in part to the differences among women in the three patient groups. Women who started HAART after pregnancy were more likely to have been previously exposed to non-HAART ART, to have started triple-NRTI-based HAART regimens, and to have started HAART during an earlier HAART era. These factors are known to lead to poorer responses to HAART [25]–[27], [30]–[32]. Additionally, high proportion of women who started HAART during pregnancy for presumed PMTCT rather than their own health. However, the better virologic and immunologic responses in the pregnant group persisted after adjusting for these factors in multivariable models and after excluding persons on triple-NRTIs. Second, improved health-related behavior during pregnancy, and resumption of unhealthy behaviors postpartum [33], [34] could explain the better virologic and immunologic responses in this group. Pregnancy also has been independently associated with greater adherence to HAART [35]–[37]. Although we were unable to directly assess for adherence, we measured the intensity of clinical care via the number of provider visits per month. Women initiating HAART during pregnancy were seen more frequently during the study period. This in turn provided a better opportunity to provide adherence counseling, which is a part of routine care of pregnant women at the Comprehensive Care Center. Improved adherence in turn could lead to virologic suppression [38], [39], prevention of ADE and death [40], [41] and of mother-to-child transmission [42]. The above factors may explain the poorer virologic and immunologic responses to HAART in women starting HAART after pregnancy. Third, the enhanced virologic and immunologic response among women who started HAART while pregnant could be related to the elevated levels of progesterone and estrogen during pregnancy, which lead to an increase in CD4+ CD25+ regulatory T cells and tolerance to alloantigens such as fetal antigens [21], [43]. Increased levels of regulatory T cells might limit CD8+ lymphocyte effector function that in turn could limit HIV-1 infection-associated immune dysregulation and reduce immune cell exhaustion and programmed cell death [44], [45]. Thus, the effect of pregnancy on regulatory T cells could possibly lead to a better virologic response to HAART among women starting first HAART during pregnancy

Due to the observational nature of this study, we cannot make causal inferences between timing of HAART initiation and HIV-related outcomes. First, our study had low power to detect differences in clinical outcomes between pregnancy groups. The number of women who started HAART before pregnancy was particularly low. Larger studies with longer follow-up may show statistically significant differences in HIV disease progression. Second, women have different indications for HAART initiation during pregnancy [2] compared to the indications when women are not pregnant [46]. Although we adjusted for important baseline characteristics that are associated with immune recovery and virologic suppression (such as baseline CD4+ lymphocyte count and HIV-1 RNA, CD4+ lymphocyte count nadir [47], injection drug use [48], [49], and hepatitis C virus co-infection [50]) residual confounding by indication for HAART initiation might still remain. Third, it should be recognized that of women who started HAART prior to becoming pregnant only those who survived were able to get pregnant at a later date and, therefore, were included in our study. Fourth, we were unable to assess and control for HIV-1 resistance that could be associated with prior non-HAART ART exposure and may contribute to poorer responses to HAART [51]–[56].

With the above noted, this study found an improved virologic and immunologic response among women who started HAART during pregnancy compared to women who started HAART after pregnancy. Regardless of the underlying etiology, it is an important finding that women initiating HAART during pregnancy had excellent virologic and immunologic responses; this presumably benefits both the mother and the fetus. Larger studies are warranted to assess for possible differences in subsequent HIV disease progression, particularly in resource-limited settings, and to investigate the factors associated with the improved outcomes during pregnancy, such as adherence or underlying biologic factors.
